# Developing Future-Ready University Graduates: Nurturing Wellbeing and Life Skills as Well as Academic Talent

**DOI:** 10.3389/fpsyg.2022.827517

**Published:** 2022-03-04

**Authors:** Tzyy Yang Gan, Zuhrah Beevi, Jasmine Low, Peter J. Lee, Deborah Ann Hall

**Affiliations:** ^1^Foundation Programmes, Heriot-Watt University Malaysia, Putrajaya, Malaysia; ^2^Department of Psychology, Heriot-Watt University Malaysia, Putrajaya, Malaysia

**Keywords:** self-reflection and evaluation, leadership, happiness and wellbeing, positive psychology, positive education outcomes

## Abstract

Higher education is starting to embrace its role in promoting student wellbeing and life skills, especially given the concerning levels of poor mental health and uncertainties in the future job market. Yet, many of the published studies evaluating positive educational teaching methods thus far are limited to interventions delivered to small student cohorts and/or imbedded within elective wellbeing courses, and are focussed on developed Western countries. This study addressed this gap by investigating the effectiveness of an institution-wide compulsory course informed by the principles of Seligman’s Wellbeing Theory. The course was delivered at a British university in a developing country in Southeast Asia. It purposefully sought to nurture growth-oriented outcomes (including self-awareness, positive emotions, and personal effectiveness) and was taken by an entire cohort of year one undergraduate students. We tested the effectiveness of the curriculum content and staff coaching style in achieving life skills, and evaluated how these perceptions influenced students’ subjective wellbeing. A convergent mixed-methods design was used with 350 survey respondents and 11 interviewees. Perceived life skills scores showed a 2.5% improvement at the end of the course. Partial Least Squares Structural Equation Modelling tested the predicted relationships between variables. All relationships were statistically significant, but the influence of course design and educators’ style on life skills acquisition (50.8% of the variance) was moderate, while the effect on subjective happiness and life satisfaction (4–5% of the variance) was very weak. Qualitative data indicated that while quantifiable benefits to wellbeing might not be immediate, students did anticipate longer-term benefits for happiness and life satisfaction. This finding suggests that such a novel educational approach is well-received by Asian students and may sow the seeds for future benefit by positively impacting on their skills, behaviours, attitudes, and values. To achieve optimal flourishing at university, we recommend exploring teaching practises that combine positive education with coaching psychology practises.

## Introduction

The COVID-19 global pandemic has accelerated a transformation in the skills required by today’s workforce, as well as in the need for adaptability to change. Research by the McKinsey Global Institute identified self-leadership and inter-personal skills as two of the four skills categories essential to future-proof those graduates emerging into the job market (Dondi et al., 2021); the others being cognitive and digital skills. McKinsey’s definition of self-leadership comprises elements of wellbeing (e.g., optimism, grit, and coping with uncertainty) and life skills (e.g., understanding one’s strengths, driving change and achievement orientation). A wake-up call to university educators from this report was the conclusion that higher education is failing to effectively develop graduates with proficiency in self-leadership skills, despite these skills being most closely associated with employment, income, and job satisfaction.

Relevant to the teaching of self-leadership is the branch of psychological theory and practise known as Positive Psychology; the science of wellbeing and flourishing (e.g., Seligman, 2011; Wong and Roy, 2017; Lomas et al., 2020). For the past 10 years or so, a number of positive psychologists have advocated teaching students wellbeing and life skills, in addition to traditional academic skills (c.f. Positive Education; Seligman et al., 2009). The goal of this positive education approach is to improve students’ wellbeing *and* learning through activities that explicitly promote components of wellbeing. Seligman’s Wellbeing Theory underlies one of the most common approaches by teaching positive emotion, engagement (i.e., being in the flow), positive relationships, meaning (i.e., purpose in life), and achievement (Seligman, 2011). While there are numerous examples of positive education in schools (e.g., Norrish et al., 2013), positive education has had fewer inroads in the higher education setting despite universities being entrusted to prepare generations of youths for a better future. There are a growing number of case studies. For example, at Melbourne University Centre for Positive Psychology, Australia, a 6-week University Wellbeing Programme for undergraduates in a Positive Psychology course led to improvements in mental health and seemed to buffer against stress, relative to a control group (Young et al., 2020). In the United Kingdom, an optional 12-week psychoeducational happiness course for undergraduates at the University of Bristol improved student mental wellbeing, even during the COVID-19 pandemic (Hood et al., 2021). However, of those studies conducted at university, many are limited to evaluating interventions delivered to small student cohorts and/or imbedded within elective wellbeing courses. Hence, their generalisability is somewhat reduced.

A number of specific challenges in implementing positive education principles within the general university curriculum have been recognised (e.g., King et al., 2016). The university environment itself may provide one such tension. Positive education requires a cultural shift away from the university as an institution of knowledge production and teaching delivery, toward one of fostering self-discovery and personal growth. To achieve those goals requires a coaching style that nurtures student potential and talent. Yet, the traditional university system emphasises one-way communication from teacher to student and independent learning (Simel and Bognar, 2017). Here, leadership from the senior management is needed to push the organisation and its staff in the right direction.

One challenge coming from within the positive education field itself concerns the geographical bias in research outputs toward Western, Educated, Industrialised, Rich and Democratic (WEIRD) countries. The reasons for this are complex (e.g., Wong, 2020). Nevertheless, this paucity of research is especially awkward given that culture is well-known to play a key role in wellbeing and learning. For example, a study of Singaporean secondary school students found that, unlike their Western counterparts, they were equally strongly motivated to study by external factors (e.g., the need to show parents that they are achieving) as well as intrinsic factors (e.g., personal satisfaction and an inherent desire to learn) (Caleon et al., 2015). The authors reasoned that this finding reflects those cultural traditions which put primacy on learning as a means for social mobility and economic survival; a characteristic that is shared by many developing countries in Asia.

Important questions therefore remain whether wellbeing and life skills can be attained in a university culture that embraces positive education across the whole institution and that attempts to offer a coaching style of teaching, and whether a curriculum informed by a WEIRD-centric conceptual framework for wellbeing can have impact in the setting of a developing Asian country. Since 2018, Heriot-Watt University Malaysia has offered students a transformative educational experience through its compulsory year one ‘EmPOWER’ programme. This coaching programme supports self-discovery and personal growth, accentuates purpose and offers opportunities to achieve a positive impact on the community and society, with achievements being explicitly articulated to future employers through an enhanced transcript.

### The Current Study

The purpose of this study was to evaluate this programme using a convergent parallel mixed-methods design, gathering quantitative online survey and qualitative interview data. We tested the hypotheses that (i) the course would improve students’ wellbeing in accordance with Wellbeing Theory, (ii) the course would improve students’ life skills (namely sense of purpose, self-reflection, self-awareness, leadership, inter-personal communication and positive emotions), in line with the intended positive education learning outcomes, (iii) that the course design and educator style would each positively influence students’ learning of those life skills, and (iv) that achievement in life skills would in turn drive greater wellbeing as measured by subjective happiness and life satisfaction. The purpose of the interview data was to give greater insight into how the positive education course influenced students’ perceptions of their learning.

## Materials and Methods

### Participants

Invited participants were all year one students (*n* = 570) enrolled in undergraduate programme at Heriot-Watt University Malaysia in the academic year 2019/20. Participants were eligible to take part in the study if their overall attendance was at least 80%. Survey data was pseudo-anonymised to enable matching responses to attendance records, and was fully anonymised through attribute suppression before data analysis. Although 407 students completed the survey, 57 were excluded because they did not meet the eligibility criterion regarding attendance: leaving 350 survey respondents at T3. From these, 104 did not complete the SHS and SWLS at T2. Analysis of the changes over time handled these missing data using a pairwise deletion method (*n* = 246).

Survey respondents were invited to volunteer for a short follow-up interview at T3 (*n* = 11). A total of 14 interviews were conducted, but data from three participants (participants 02, 06, and 14) were later removed due to technical difficulties with the recording which rendered a fair transcription impossible. No information about gender was collected for the quantitative survey data, since this was not deemed relevant to the hypotheses. For the qualitative interview, the 11 included participants were six male and 5 female students enrolled in a variety of programmes (Psychology, Finance and accounting, Business, Actuarial science, and Engineering).

### Instruments

**FIGURE 1:**
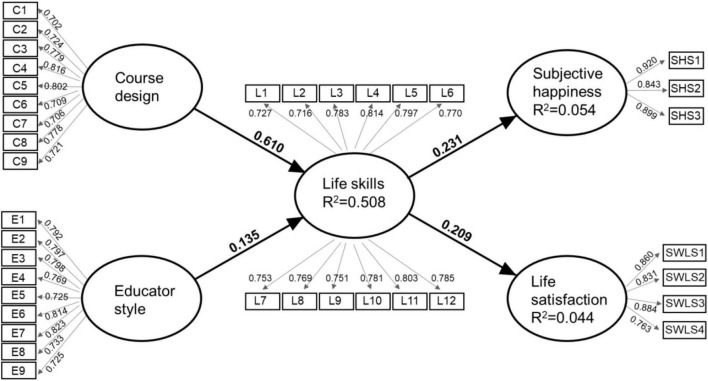
Reflective measurement model and structural model. The latent variables are the predictors (circles), and the corresponding measurement items are the outcomes (rectangles). The model accounts for measurement error at the item level (not shown).

Prior to data analysis, a structural model was constructed from a review of the published literature, indicating the latent and measured variables, and their causal relationships (Figure 1). The central part of the structural model is founded on the premise that the effectiveness by which a student achieves *life skills* as measured by the positive education learning outcomes, are determined by an interplay between factors, which can be classified primarily as the *course design* and *educator style* (McKone, 1999; Eom and Ashill, 2016). In the context of the higher education setting, we predict that the degree to which a student perceives that (s)he has achieved these life skills may in turn influence their emotional state and sense of optimal wellbeing (Seligman et al., 2009) which we operationalise within the structural model as *happiness* and *life satisfaction*, respectively. The full measurement model is shown in Figure 1. It is a reflective measurement model since the indicators of each construct are considered to be caused by the construct itself and the items are interchangeable.

The five latent variables are shown in Figure 1 (measurement scales are reported in Supplementary Table 1). Course design was measured using a set of nine tailor-made statements which asked about the content (C1-3), learning objectives (C4), structure (C5), level of enjoyment (C6), and assessment (C7-9). Educator style was measured using another set of nine tailor-made statements which asked about educator’s personal characteristics and attitudes (E1, E4-5), feedback (E2-3), knowledge (E6-7), and inter-personal skills (E8-9). Life skills were measured using a set of bespoke statements that were directly informed by the course learning objectives. Statements covered each of the three major taxonomic categories of student learning; knowledge (L1), skills (L2-3, L6, L9-10, and L12), and attitudes (L8, L11), plus behaviour (L4-5, L7). All three sets of questions on course design, educator style and life skills had a forced four-point Likert scale (*Strongly disagree* to *Strongly agree*). There was no safe ‘neutral’ option which is sometimes seen as an easy option to take when a respondent is unsure, and so may not reflect a truly neutral point of view (Allen and Seaman, 2007).

Global subjective happiness was measured using the unidimensional “Subjective Happiness Scale” (SHS, Lyubomirsky and Lepper, 1999). Developed and validated in the United States, the SHS considers happiness from the respondent’s own perspective and therefore accounts for differences in cultural expectations between Western and Asian societies. The original scale has four statements, but we removed “Some people are generally not very happy. Although they are not depressed, they never seem as happy as they might be. To what extent does this characterisation describe you?” because the counterpart statement about happiness (item 3) was deemed sufficient. The response format was a seven-point Likert scale, with higher scores reflecting greater happiness. We defined life satisfaction as the cognitive assessment of one’s life as a whole. Individuals tend to use their own benchmarks and criteria in making this assessment and so it is more meaningful to assess global judgements of life satisfaction, rather than satisfaction with specific life domains (Diener and Diener, 1995; Proctor et al., 2009). Students’ life satisfaction was measured using the unidimensional “Satisfaction With Life Scale” (SWLS; Diener et al., 1985). Developed and validated in the United States, it has been validated in Asia (Bai et al., 2011). The original scale has five statements, three referring to satisfaction with the present, and two to satisfaction with the past. We removed “If I could live my life over, I would change almost nothing” since this was deemed to have inadequate construct validity for our target youth sample. Responses were indicated using a seven-point Likert scale (*Strongly disagree* to *Strongly agree*), and the global score has previously been shown to be sensitive in discriminating the full range of life satisfaction from extremely dissatisfied to extremely satisfied (Pavot and Diener, 1993). We are justified in removing individual items from the SHS and SWLS since in the context of the reflective measurement model, all items are expected to be correlated (i.e., possess internal consistency reliability), and so dropping an item from the measurement model should not alter the meaning of the items (Jarvis et al., 2003).

### Procedure

The positive education lectures and workshops were conceived by the senior management team, compulsory for students and formed part of a credit-bearing “Self-Empowerment and Social Responsibility” course that constituted level 1 of the EmPOWER programme. This 24-week course was designed with expert input from educators and a practising consultant in positive psychology. The curriculum was informed by Seligman’s Wellbeing Theory (Seligman, 2011), incorporating the principles of positive psychology by blending development in skills that promote thriving (e.g., self-awareness, intrinsic motivation, and leadership; Benson and Scales, 2009), with an emphasis on developing by mapping personal strengths and opportunities (Sprangel et al., 2011), character strengths and virtues (Peterson and Seligman, 2004). The curriculum also included discovering purpose and creating a plan to mobilise that purpose into positive impact (Benson and Scales, 2009). Each week had 2 h of contact time, comprising conventional lecture-style presentations plus workshops to develop a personal impact statement comprising three personalised statements “I am a …. My purpose is …. I will …..” Additional personal study time was required to complete an impactful community project through team-working and homework exercises that required self-reflective practise.

The study was approved by the Social Sciences Ethics Committee Heriot-Watt University on September 2019 (ref: 2019-120), and all participants gave online (survey) and verbal and written (interview) informed consent in April 2020. Quantitative data were gathered through an online survey using Qualtrics software (Seattle, United States). Course design, educator style, and life skills were measured at T2 and T3. Two of the life skills (items L11: positive thoughts, and L12: critical thinking) were assessed only at the end of the course (T3) because they had not yet been adequately covered at the mid-way point. SHS and SWLS were assessed at three time points: T1 - prior to the start of the course, T2 – at the mid-way point (12 weeks), and T3 – at the end of the course (24 weeks).

Qualitative data were gathered through a semi-structured interview, conducted by ZB, one of the co-authors and a health psychologist, who was not involved in the delivery of the course. Questions probed how the life skills were achieved. The interview followed the approach of Interpretative Phenomenological Analysis which enables multiple participants who experience similar events to tell their stories without any distortions and then seeks to make sense of the ‘lived experiences’ of the research participants (Alase, 2017).

### Design and Analysis

Change over time in SHS and SWLS were examined using a one-way ANOVA, and change over time in life skills were examined using a paired *t* test.

For the quantitative survey data, the hypothesised structural model in Figure 1 was analysed using Partial Least Squares Structural Equation Modelling (PLS-SEM) implemented with Smart PLS version 3 (SmartPLS GmbH, Germany). PLS contains a two-step procedure as recommended by Henseler et al. (2009), which involves the evaluation of the (outer) measurement model followed by evaluation of the (inner) structural model.

The assessment of the measurement model (Figure 1) used the T3 data (*n* = 350) and involved examining the reliabilities of the individual items (indicator reliability), internal consistency [Cronbach’s alpha and composite reliability), convergent validity (Average Variance Extracted (AVE)] and discriminant validity (Fornell-Larcker criterion, heterotrait-monotrait (HTMT) ratio of correlations criterion, cross-loading; Fornell and Larcker, 1981; Hair et al., 2017).

As a measure of indicator reliability, the factor loadings of the measurement items should be higher than 0.70 for inclusion and should be deleted if less than 0.40 and 0.70 (Hulland, 1999; Hair et al., 2017). Regarding internal consistency in the scale items, Cronbach’s alpha (α) is a measure of internal consistency (i.e., how closely related the observed items were in representing the underlying construct), while Composite Reliability (CR) is an indicator of the shared variance among the observed items used as an indicator of a latent construct. For good reliability, α should be greater than 0.70 and CR should be 0.70–0.95 (Hair et al., 2017).

Convergent validity assesses whether the actual results confirm expected relationships between items and underlying constructs. The Average Variance Extracted (AVE) measures the level of variance captured by a construct vs. the level due to measurement error, and a value of 0.50 is acceptable while 0.7 is very good (Fornell and Larcker, 1981). Discriminant validity assesses the extent to which the underlying constructs in the model are distinct from one another (Hulland, 1999). To satisfy this requirement, items should load more onto their intended construct than on another. This requirement was tested by three indices (Hair et al., 2017). Under the Fornell-Larcker criterion, the square root of each construct’s AVE should have a greater value than the variance of the other constructs. The HTMT value should be lower than 0.85 (Kline, 2011), although some authors suggest 0.90 (Teo et al., 2008). And each measurement item should correlate weakly with all other constructs, except for the one to which it is theoretically associated, which should be higher than 0.70 (Hair et al., 2017).

In step two of the PLS approach, we evaluated the structural model’s predictive ability and the relationships between the constructs. The coefficient of determination (R^2^), path coefficients (β), and *t*-statistic values, effect size (f^2^) and the predictive relevance (Q^2^) are the key standards for such evaluation (Hair et al., 2017). R^2^ measures the amount of variance explained in the endogenous constructs (i.e., life skills, happiness and life satisfaction). Following Hair et al. (2017), *R*^2^ = 0.75 is considered substantial, *R*^2^ = 50 is regarded as moderate, and *R*^2^ = 0.26 is considered weak.

The path coefficients (β) represent the strength of the hypothesised relationships between the constructs. A bootstrapping technique with 5,000 resamples was conducted to estimate the beta (β) and corresponding *t* values as recommended by Chin et al. (2003). The greater the beta coefficient (β), the stronger the effect of an exogenous construct on the endogenous construct. Path coefficients with a value close to 1 represents a strong positive relationship, and conversely a value closer to −1 represents a strong negative relationship. The overall effect size (f^2^) is a measure of the degree of impact of the path relationship. Following Hair et al. (2017), f^2^ = 0.75 is considered large, f^2^ = 0.15 is regarded as medium, and f^2^ = 0.02 is considered small. The predictive relevance for the structural model was evaluated using Q^2^ (Tenenhaus et al., 2005) and so it can be considered an indicator of the quality of the structural model. Interpretation of Q^2^ followed that of Hair et al. (2017), with a value > 0 indicating adequate predictive relevance, and a value < 0 indicating poor predictive relevance.

For the qualitative interview data, we employed a Framework Method to analyse the anonymised interview transcripts. The Framework Method is most commonly used for the thematic analysis of semi-structured interview and it is appropriate for application to reasonably homogenous data covering similar topics (Gale et al., 2013). Our approach was partly deductive because coding proceeded through the lens of predefined categories based on the structural model: (i) how the course content influenced perceived life skills, (ii) how the instructor style of delivery influenced students’ learning, (iii) happiness, and (iv) life satisfaction. Coders followed the Framework Method steps (i.e., Transcription; Familiarisation with the interview; Open coding; Developing an analytical framework by structuring the codes under each category; Applying the analytical framework to the remaining transcripts; Charting the data into the framework matrix; Interpreting the data), and used Quirkos version 2.4.2 (Quirkos, Scotland). Two coders (ZB and DH) who were not part of the EmPOWER teaching team independently completed the open coding. Open coding was first conducted on three transcripts to confirm that important aspects of the data were not missed by restricting to these four categories. Data interpretation interrogated the theoretical concepts (either prior concepts or ones emerging from the data) and mapped connections between categories to explore relationships or causality.

## Results

Data from the 246 included respondents did not support hypothesis (i) that the course would improve students’ wellbeing in accordance with Wellbeing Theory (Seligman, 2011). There was no detectable change in wellbeing as measured by the SHS [*F*(2,490) = 1.317, *p* = 0.269] and SWLS [*F*(2,490) = 0.363, *p* = 0.696] (Table 1 and Supplementary Table 2).

**TABLE 1:** Scores on survey data assessed over the duration of the course.

Measure	T1 M (SD)	T2 M (SD)	T3 M (SD)	Statistic	*p* value
Life skills L11 (Positive thoughts) L12 (Critical thinking)	– – –	28.19 (4.40) – –	29.17 (4.28) 2.92 (0.50) 2.96 (0.54)	−3.741	< 0.001
Subjective happiness	14.31 (3.40)	14.18 (3.21)	14.47 (3.29)	1.317	0.269
Satisfaction with life	18.73 (4.73)	18.59 (4.49)	18.52 (4.53)	0.363	0.696

*T1, pre-intervention; T2, mid-way point; T3, post-intervention. Subjective happiness scores range from 3 to 21. Satisfaction with life scores from 4 to 28. Life skills scores range from 10 to 40.*

As the course progressed from T2 to T3, there was a small, but statistically significant increase in life skills scores [*t*(245) = −3.741, *p* < 0.001] (Table 1 and Supplementary Table 2). Thus, hypothesis (ii) was supported.

Directional influences between variables (i.e., hypotheses iii and iv) were evaluated using Structural Equation Modelling. The (outer) measurement model defined in Figure 1 met all *a priori* evaluation criteria regarding the reliability and validity of the items in the constructs (Table 2). The factor loadings of the measurement items ranged between 0.702 and 0.920 which meets the threshold for indicator reliability and confirms the relative importance of each observed item to the underlying construct. The α values for all construct factors were 0.871–0.938 and CR values were 0.902–0.946, indicating the scales had acceptable reliability. AVE values were acceptable for course design, educator style and life skills (i.e., 0.562–0.602) and very good for happiness and life satisfaction (i.e., 0.699–0.788), and so convergent validity was confirmed for this hypothesised model.

**TABLE 2:** Indicator reliability and internal consistency.

Main constructs	Items	Loadings	α	CR	AVE
	C1	0.702			
	C2	0.724			
	C3	0.779			
Course design	C4	0.816	0.903	0.920	0.562
	C5	0.802			
	C6	0.709			
	C7	0.706			
	C8	0.778			
	C9	0.721			
	E1	0.792			
	E2	0.797			
	E3	0.798			
Educator style	E4	0.769	0.917	0.931	0.602
	E5	0.725			
	E6	0.814			
	E7	0.823			
	E8	0.733			
	E9	0.725			
	L1	0.727			
	L2	0.716			
	L3	0.783			
	L4	0.814			
	L5	0.797			
Life skills	L6	0.770	0.938	0.946	0.595
	L7	0.753			
	L8	0.769			
	L9	0.751			
	L10	0.781			
	L11	0.803			
	L12	0.785			
	SHS1	0.920			
Happiness	SHS2	0.843	0.871	0.918	0.788
	SHS3	0.899			
	SWLS1	0.860			
Life satisfaction	SWLS2	0.831	0.856	0.902	0.699
	SWLS3	0.884			
	SWLS4	0.763			

*For details of the items see Supplementary Table 1. A, Cronbach’s α; CR, composite reliability; AVE, average variance extracted.*

The model also met all *a priori* evaluation criteria regarding the discriminant validity of the latent constructs. From Table 3, it can be seen that the square root of each construct’s AVE for each construct was greater than the correlation involving the constructs thus meeting the criterion of Fornell and Larcker (1981), and from Table 4 the results also passed the HTMT criterion test with values not exceeding 0.85. Moreover, from the cross-loading evaluation all the loading indicators on the assigned construct were > 0.70 and higher than the loadings on the other constructs (Supplementary Table 3).

**TABLE 3:** The Fornell-Larcker Criterion Test for discriminant validity which requires values in bold to be greater than the remaining values in each column.

	Course design	Educator style	Life skills	Happiness	Life satisfaction
Course design	**0.750**				
Educator style	0.713	**0.776**			
Life skills	0.707	0.570	**0.771**		
Happiness	0.210	0.210	0.231	**0.888**	
Life satisfaction	0.202	0.240	0.209	0.564	**0.836**

**TABLE 4:** The Heterotrait-Monotrait (HTMT) Criterion Test for discriminant validity which requires values not to exceed 0.85.

	Course content	Educator style	Learning outcomes	Happiness	Life satisfaction
Course design					
Educator style	0.782				
Life skills	0.749	0.606			
Happiness	0.222	0.220	0.237		
Life satisfaction	0.232	0.269	0.230	0.645	–

Based on these findings, the proposed structural model was accepted, with confirmation of adequate reliability, convergent validity, and discriminant validity. The next step was to evaluate the (inner) structural model outcomes comprising the latent constructs and path coefficients (Figure 1).

Hypothesis (iii) predicted that the course design and educator style would each positively influence students’ learning of life skills, and this was supported by our data. Course design contributed in a meaningful way to learning life skills, while the educator style played only a small positive role. The R^2^ value for life skills is 0.508 meaning that the two exogenous constructs (course design and educator style) explain 50.8% of the variance in this endogenous construct (Figure 1). This is a moderate effect (Hair et al., 2017). The Q^2^ value for the life skills construct was equal to 0.290, which was higher than the threshold limit, indicating that the path model had sufficient predictive relevance for this construct (Hair et al., 2017). From the path coefficients, both course design and educator style significantly and positively influenced life skills, β = 0.610 and 0.135, respectively, *p* < 0.05 (Table 5). Considering the effect size for the influence of course design on life skills (f^2^ = 0.372, Table 5), the result exceeded a ‘medium’ effect (Hair et al., 2017). In contrast, for educator style, f^2^ was 0.018 which is considered small.

**TABLE 5:** Path coefficients, *t* statistics, and effect size (f^2^).

Hypothesised path	Std β	Std error	*t* statistic	*p* value	f^2^
Course design → Life skills	0.610	0.065	9.401	0.000	0.372
Educator style → Life skills	0.135	0.068	1.983	0.024	0.018
Life skills → Happiness	0.231	0.056	4.161	0.000	0.057
Life skills → Life satisfaction	0.209	0.052	4.038	0.000	0.046

Hypothesis (iv) predicted that achievement in life skills would in turn drive greater wellbeing as measured by subjective happiness and life satisfaction. Statistically speaking, the hypothesis was supported, but the size of the influence was very small. The smaller R^2^ values for happiness (0.054) and life satisfaction (0.044) suggest that life skills explain very little of the variance in these endogenous constructs (i.e., 5.4 and 4.4%, respectively) (Figure 1). While the Q^2^ values for the happiness and life satisfaction constructs also reached sufficient predictive relevance (0.036 and 0.027, respectively), they were only just greater than 0, indicating barely adequate predictive relevance (Hair et al., 2017). From the path coefficients, life skills significantly and positively influenced students’ self-reported happiness and life satisfaction, β = 0.230 and 0.209 respectively, *p* < 0.001 (Table 5). However, the corresponding effect sizes were small (Hair et al., 2017).

### Understanding the Lived Experience of the Learning Process

The interview findings shed light on the paths influencing perceived life skills acquisition. Four themes emerged about the course design. It was students’ first opportunity to engage in life skills training, it gave them a chance to demonstrate their ability to articulate what they had learned, the course instilled a genuine sense of excitement to learn, and it promoted a greater self-awareness and sense of purpose. Three themes emerged about educator style. Learning activities were engaging and informative and students were encouraged to apply their knowledge, but there were some criticisms about quality of the teaching staff. No themes on happiness were observed, but there was some evidence that the course contributed to life satisfaction as it encouraged students to engage in cognitions and behaviours that would promote success.

#### Course Design

For many interviewees, this was the first time they had engaged in self-reflection to know themselves better and in life skills training to develop personal effectiveness. Moreover, interviewees recognised and appreciated unique opportunities open to them that had not been offered at school nor were available to their friends at other universities.

• Student 1 said: “*I kind of enjoy this because. In our public school system in our country we don’t really have something like this. So to come to university and to see something like this, it’s something that I appreciate, I can appreciate that personally*…. *When I first saw that there was a course like this, I was personally quite delighted because this is based off my previous experiences in other universities, they don’t really put that much emphasis on programmes like this.”*
• Student 3 said: “*So I told them [my friends]” and their response was “Wow, it’s a very good initiative.” My friends are from other university, and they mentioned that there is a lack of talks and all those stuff. And they are surprised to see, “Wow, you really have a programme that motivates you to attend those talks.”*
• Student 5 said: *“All these project managing, working as a team, all our leadership skills, other universities do not have that.”*
• Student 12 said: *“Cause before I joined the university [*…*] I was probably a bit lost in several ways. But during the EmPOWER program, they introduced us to some concepts and some personality traits that I’ve never really think about before. So I think that really helps me reflect how a person I am, in what way.”*

Students were also able to articulate how they had developed certain personal effectiveness skills, particularly leadership (Learning outcome L2), team-working and communication (L6) and time management (L7).

• Student 3 said: *“I have a good leadership skill*…*I try to improve myself, like joining those clubs and societies, so that when I put it in the LinkedIn post*…*so people will get to notice you, your leadership skills.”*
• Student 4 said: ‘*I think it’s a really good platform for me to start leading people such as. I pick up the responsibility of saying, “Okay, I will do this. I will lead the group. I will delegate a task and then communicate with my members so that my members would say.without a leader in the group, I think everything would be chaos. I took the initiative to become a leader. And because of that, I think it helps me to improve my leadership skills. I have definitely improved on my time management skills, because we managed to finish everything on time. We were actually ahead of schedule. And despite certain setbacks from the conflict, we actually still managed to get back ahead of schedule also.”*
• Student 13 said: *“Toward the end, before we conduct our social project, we got into a little bit of a disagreement and then to solve that, we did it as team, so I think that was a good experience ’cause everyone set aside our differences and we got together to solve something.”*

Students were also able to articulate how they had developed greater emotional awareness (Learning outcome L3), self-regulation (L4), ability to express gratitude (L8) and ability to reframe thoughts more positively (L11).

• Student 4 said: *“I think one of the weakness. My last one was actually something about love. And I do not know how to harness that in my networking among my university friends and lecturers. But maybe I would just say that, through these few months I was in uni, maybe I would say I will just increase. Further strengthen my love toward myself and then my love toward friendships, friendships and professional development.”*
• Student 5 said: *“A positive impact it did for me is that, after writing the gratitude letter, it kind a opened my eyes to everything else that I should be grateful for.”*
• Student 7 said: *“I’m not usually the type to show love, but because of the gratitude letter, it helps me to show more love to people, so it did help me also.”*
• Student 8 said: *“It was really good. I actually did it for my grandfather, who has recently passed away, so. He was happy. He was very happy. I’m really glad I did it.”*

The course instilled a genuine sense of excitement to learn.

• Student 5 said: *“I guess, my highlight [*…*] was the Community Project itself, because it kinda opened my eyes to [*…*] how work will be, it kinda gives this. forces us to work as a team or work on our own, so we kinda have an idea on how we can apply certain things later on.”*
• Student 9 said: *“I remember the attendance was also many because all of us was very excited and interested to hear this workshop.”*

Interviewees mentioned on a number of recurring aspects of the course design about how the content had enabled them ‘.*to develop our personal skill which we cannot learn during lectures’* (Student 10). The purpose work (Learning outcome L1) left a lasting impression. Overall, the purpose activities instilled a greater sense of who students are and where they are heading, particularly at a formative time in year 1 when they had further years of study ahead.

• Student 3 said: *“Impact statement, what I find it about is, it’s very interesting because even. It knows what you wanna achieve in the future*… *I think that it helps to know what you’re capable of in the future, it helps to realise what are your goals, what are your dreams, what you wanna achieve*.”
• Student 7 said: *‘It helped me a bit because before the impact statement, I don’t know what actually am I doing. I’m just doing accounting because I thought this is the easiest for me. But with the impact statement, at least I have a purpose*…’
• Student 12 said: *“EmPOWER course is very helpful for especially year one students, who, some of them just entered the proper university environment. And it really helped students to find their purpose and set their goals during the 3–4 years of study in university. And I think it also helps to encourage students to think more about themselves and to really find what kind of person they are.”*

#### Educator Style

The active and positive style of delivery played a contributory role in making the learning activities engaging and informative and encouraging the students to learn new things (Learning outcome L10).

• Student 9 said: *“They reach out to us very often, and remind us on the task, and then it’s like more, how to say, closer to us. We can feel that they are very genuinely want to help us, to improve.”*
• Student 12 said: *“Let’s say the workshop facilitators were very empowering. They were very active. And also, they really help us to follow the instructions and progress through the workshops smoothly.”*
• Student 13 said: *“I would say that the instructor was a really helpful person firstly, he gave us certain points, like what to do in this section? What to put in this section? Why it’s working and why is not? And all that. I think that was always useful. And he also told us dos and don’ts.”*

The hands-on activities encouraged students to apply what they had learned, and this reinforced all life skills that were taught.

• Student 7 said: *“And then we need to do a project like how to clean the river. So actually we did. Like a hands-on activity there because we discuss in the group how to do. How to build something to clean the river. She did the lecture for 1 h and she gave us like 30 min to do the project and another 30 min for presentation. So for me, that is quite interesting because we don’t actually sit there for 2 h straight. We’re actually like doing something and we’re actually using the techniques that she’s giving.”*

Not all aspects of teaching were well received, and some students commented on the variability in quality of the teaching staff. This may have affected the model findings, particularly the relationship between educator style and life skills.

• For example, student 7 recognised that that not all workshops embedded interactive activities: “*I would prefer it will be like 1 h lecture and another 1 h for us to use that skill, like we try it ourselves.”*
• Student 4 said: “… *in all honestly, because they are just reading from the slides, to be honest [*…*] instead of maybe giving lectures and stuff, like giving talks for 2 h, you should give something more hands-on, more activities, something that requires thinking*.”
• Student 1 said: “*Diversifying the power speakers is a good idea [*…*] But the problem with that is consistency [*…*] They don’t deliver it to that similar standard that we come to have expected from the previous week.*”

#### Life Satisfaction

No themes on happiness were observed, but there was some evidence that the course contributed to life satisfaction as it encouraged students to engage in cognitions and behaviours that promote success. For example, student 13 mentioned: *“I would say that it’s worth the shot. I would recommend it*…*it’s interesting to know that there are certain things that you think you know yourself, but you don’t really know. You don’t know the kind of potential that you could unleash. So this programme kinda helped me to find my potential and things that I could do better or improve on.”* Student 7 also reflected on how the life skills training made them reflect on what was important in their life to achieve personal satisfaction. *“I think more programmes about why. I mean why we need to help more people, something like that, instead of focussing on your work, instead of focussing like to achieve. Instead of focussing to achieve money and stuff, maybe we can say that there’s more to life like we need to be like a good people, so everyone can enjoy life together, I think. Yeah, because for me money is everything, but at the same time, life. Like to help others is more important for me.”*

## Discussion

To our knowledge, this is the first report of a holistic development programme conceived by the senior managerial team, fully integrated into the curriculum and completed by all university students enrolled across an academic year group. Moreover, the study was conducted in an Asian setting. The principal finding from the quantitative data demonstrated improved life skills, mostly achieved through the course design. The qualitative data added value to this finding by illustrating how learning was not limited to knowledge acquisition, but that the students recognised what psychosocial skills, attitudes and values are needed for positive behaviour change to occur (c.f. UNICEF, 2012).

Although there was no detectable change in subjective happiness and life satisfaction scores over time, perceived life skills did exert a small influence on wellbeing at the end of the course. The qualitative data indicated that important seeds had been planted which could promote a mindset for flourishing in adulthood. Longitudinal studies have shown that positive education interventions are at their best when they exert measurable benefits that evolve over time, such as by enhancing academic application through greater engagement in the learning process (e.g., Denovan et al., 2020). In their systematic narrative review, Nasheeda et al. (2019) concluded that many life skills programmes in developing countries (including those across Southeast Asia) fail to achieve sustained benefits because they focus on one-shot or short-term interventions rather than on ongoing activities. The same criticism can also be levied at positive education programmes across Asia (Hall et al., 2021a). The programme under study here is intended to foster lifelong impact as students progress through the levels of the EmPOWER programme throughout their student journey. Although our study design did not include a long-term follow-up assessment, we would recommend testing intervals over the course of the student journey to examine the learning trajectory. This would require institutional resource for systematic planning, implementation, monitoring and evaluation (UNICEF, 2012).

From our cross-sectional modelling data that assessed short-term effects, we conclude that effective course content is more critical than educator style for good life skills acquisition. This finding is consistent with the published educational literature. For example, course design is known to positively influence students’ perceived learning (Eom and Ashill, 2016), with high levels of interaction connecting students with their instructors, the course content, and their fellow classmates (McKone, 1999; Boling et al., 2012). In many ways, course design and educator style should be mutually enhancing, yet in the present study, educator style had a relatively small effect on life skills. We suggest that this may be explained by the somewhat variable student experience that was evident from the interviews, for example in the quotes cited from students 1, 4 and 7. In their exploration of thriving in adolescence, Benson and Scales (2009) reflected on the importance of the interaction between the student and the educational environment. They conceptualised that the process of adolescent thriving is animated by a passion for, and the exercise of action to nurture, a self-identified interest, skill, or capacity. Here, they proposed the role of the educator is to offer, find and create opportunities for the young person to express their spark, as well as to know, affirm, celebrate and guide that spark. Hagenauer and Volet (2014) also argued for a wider research agenda on teacher-student relationships as a relevant construct in higher education. In the context of the present study, with many hundreds of students in the class and only a small number of educators, we suggest that may have hindered the ability to create an optimal positive developmental context, even with the most passionate educators. To foster such development, several students talked about the importance of being taught by academic staff whom have the skills and competencies to facilitate an interactive learning environment that enables them to put into practise those life skills. In this regard, our results address the research gap identified by Nasheeda et al. (2019) by defining what is needed to foster optimal positive change. However, further research is needed to evaluate the impact of this recommended change in practise.

### Limitations

A large number of 570 students who were enrolled on this course did not contribute data to the study. Overall, 220 (38.6%) had an overall attendance record below the prerequisite 80%, and so they did not meet the study inclusion criteria. Hypotheses (i) and (ii) were tested using data from only 246 students (43.2% of the total sample) since 104 students attended 80% of the course, but did not complete the life skills survey, SHS and SWLS at T2. Attrition bias can weaken external validity, meaning that the study findings cannot be generalised to other populations, especially if those missing data came from students with a systematic pattern of characteristics (such as being less motivated or engaged in the course). Potentially attrition bias can also weaken internal validity, meaning that the relationships between the latent variables may change. We would argue that this is unlikely because hypotheses (iii) and (iv) were tested using data from 61.4% of the total sample (350 students) collected at T3.

### Future Research Directions

From our work, we make two recommendations to advance the field; first to conduct studies that assess long-term impact as well as short-term effects and second to understand how to maximise benefit for students, including those in Asia. In his recent editorial on the state of Positive Education in Asia, King et al. (2016) identified a lack of attention to the socio-cultural context as another important gap in the research literature. Two-thirds of the world’s population lives in Asia, yet most of the academic work is conducted in Western countries. This geographical bias is especially awkward given that culture is well-known to play a key role in wellbeing and learning (e.g., Stankov, 2013; Caleon et al., 2015). Many of the developing countries in Asia have traditionally put primacy on learning as a means for social mobility and economic survival which may explain why an Asian higher education tends to emphasise academic achievement and professional qualifications (Lin et al., 2018). Our qualitative data indicated students’ appreciation of the opportunity to address wellbeing and life skills in the context of their own learning. They also appeared to be aware that the course they were studying offered something unique compared to other education providers in the country. Other researchers have shown that youths from Asian societies have a stronger sense of family obligation compared to their Western counterparts (King and Ganotice, 2015). In contrast to the work by Caleon et al. (2015), we found no evidence for a role of external motivators such as needing to show parents that they are achieving. Instead, we found many more examples of an intrinsic motivation to learn. These findings highlight the need for more contemporary research on the Gen Z student population so that researchers and educators can better understand changing perceptions and expectations. For those educators and researchers in Asia, our work also poses the cross-cultural issue about whether families of Gen Z students are becoming more like their Western counterparts in terms of their educational expectations and career aspirations. Based on our experience with the EmPOWER programme, Asian students are calling for a shift toward a more collaborative and empowering approach to teaching (see also Hall et al., 2021b). In this regard, we may move even closer to achieving optimal flourishing by combining positive education with coaching psychology practises (Palmer and Whybrow, 2008; Seligman and Adler, 2018).

### Conclusion

The teaching of wellbeing and life skills in higher education provides students with valuable personal resources for growth. Our quantitative and qualitative research findings indicate that such a novel educational approach is well-received by Asian students and may sow the seeds for future benefit by positively impacting on their skills, behaviours, attitudes, and values.

## Data Availability Statement

The original contributions presented in the study are included in the article/Supplementary Material, further inquiries can be directed to the corresponding author.

## Ethics Statement

The studies involving human participants were reviewed and approved by Social Sciences Ethics Committee Heriot-Watt University on September 2019 (ref: 2019-120). The patients/participants provided their written informed consent to participate in this study.

## Author Contributions

TG and ZB designed the study. TG organised the database. TG and DH performed the quantitative analysis. ZB and DH performed the qualitative analysis. TG, ZB, and DH wrote the first draft of the manuscript. JL, PL, and DH revised it critically for important intellectual content. All authors contributed to conception of the study, and read and approved the submitted version.

## Conflict of Interest

The authors declare that the research was conducted in the absence of any commercial or financial relationships that could be construed as a potential conflict of interest.

## Publisher’s Note

All claims expressed in this article are solely those of the authors and do not necessarily represent those of their affiliated organizations, or those of the publisher, the editors and the reviewers. Any product that may be evaluated in this article, or claim that may be made by its manufacturer, is not guaranteed or endorsed by the publisher.
