# Supplementary material for: Developing Future-Ready University Graduates: Nurturing Wellbeing and Life Skills as Well as Academic Talent
Source: Front Psychol. 2022 Mar 4;13:827517. doi: 10.3389/fpsyg.2022.827517 (PMC8931502; doi:10.3389/fpsyg.2022.827517)
Supplement: Supplementary file 2 [file Table_2.DOCX]

**Supplementary Material 1.** The measurement scales assessing each of the five constructs in the conceptual model.

| Labels | Measurement items |
| --- | --- |
| **Course design** | |
| C1 | The EmPOWER course provides learning materials which are easy to understand |
| C2 | The EmPOWER course provides complete learning materials |
| C3 | The EmPOWER course provides relevant learning materials |
| C4 | The EmPOWER course provides clear learning objectives |
| C5 | The EmPOWER course is organised in a logical manner |
| C6 | The EmPOWER course is enjoyable |
| C7 | The EmPOWER course provides a variety of ways to assess my learning |
| C8 | The EmPOWER course provides assessments which are related to the learning objectives |
| C9 | The EmPOWER course provides clear information about the assessments |
| **Educator style** | |
| E1 | The instructors are responsive to students' concerns |
| E2 | The instructors provide timely feedback to students |
| E3 | The instructors provide helpful feedback to students |
| E4 | The instructors are enthusiastic in teaching |
| E5 | The instructors care about my individual learning |
| E6 | The instructors are knowledgeable about the course content |
| E7 | The instructors have a good understanding of the course materials |
| E8 | The instructors have good facilitation skills |
| E9 | The instructors encourage students’ interaction in the course |
| **Life skills** | |
| L1 | I am able to define my life impact statement after taking the EmPOWER course |
| L2 | I am more aware of my leadership style after taking the EmPOWER course |
| L3 | I am able to improve my emotional awareness skills after taking the EmPOWER course |
| L4 | I am able to regulate my emotional reaction after taking the EmPOWER course |
| L5 | l am able to live a more active and healthier lifestyle after taking the EmPOWER course |
| L6 | I am able to improve my communication skill after taking the EmPOWER course |
| L7 | I am able to manage my time more effectively after taking the EmPOWER course |
| L8 | I am able to express gratitude easily after taking the EmPOWER course |
| L9 | I am able to think more creatively after taking the EmPOWER course |
| L10 | I am able to learn new things with practice and effort after taking the EmPOWER course |
| L11 | I am able to reframe my thoughts more positively after taking the EmPOWER course |
| L12 | I am able to practise critical thinking in making decisions after taking the critical thinking workshop in the EmPOWER course |
| **Subjective happiness** | |
| SHS1 | In general, I consider myself: ‘Not a very happy person’ - ‘A very happy person’ |
| SHS2 | Compared to most of my peers, I consider myself: ‘Less happy’ - ‘More happy’ |
| SHS3 | Some people are generally very happy. They enjoy life regardless of what is going on, getting the most out of everything. To what extent does this characterization describe you?: ‘Not at all’ - ‘A great deal’ |
| **Life satisfaction** | |
| SWLS1 | In the most ways my life is close to my ideal |
| SWLS2 | The conditions of my life are excellent |
| SWLS3 | I am satisfied with my life |
| SWLS4 | So far, I have gotten the important things I want in life |
