# Supplementary material for: Developing Future-Ready University Graduates: Nurturing Wellbeing and Life Skills as Well as Academic Talent
Source: Front Psychol. 2022 Mar 4;13:827517. doi: 10.3389/fpsyg.2022.827517 (PMC8931502; doi:10.3389/fpsyg.2022.827517)
Supplement: Supplementary file 3 [file Table_3.DOCX]

**Supplementary Material 2.** Cross-loadings

|  | **Course design** | **Educator style** | **Life skills** | **Happiness** | **Life satisfaction** |
| --- | --- | --- | --- | --- | --- |
| C1 | **0.702** | 0.574 | 0.414 | 0.151 | 0.182 |
| C2 | **0.724** | 0.546 | 0.467 | 0.234 | 0.228 |
| C3 | **0.779** | 0.543 | 0.507 | 0.174 | 0.148 |
| C4 | **0.816** | 0.556 | 0.510 | 0.122 | 0.109 |
| C5 | **0.802** | 0.604 | 0.622 | 0.175 | 0.182 |
| C6 | **0.709** | 0.487 | 0.644 | 0.126 | 0.092 |
| C7 | **0.706** | 0.489 | 0.527 | 0.137 | 0.162 |
| C8 | **0.778** | 0.518 | 0.572 | 0.172 | 0.131 |
| C9 | **0.721** | 0.505 | 0.400 | 0.132 | 0.157 |
| E1 | 0.588 | **0.792** | 0.383 | 0.176 | 0.233 |
| E2 | 0.526 | **0.797** | 0.392 | 0.165 | 0.228 |
| E3 | 0.536 | **0.798** | 0.419 | 0.153 | 0.194 |
| E4 | 0.473 | **0.769** | 0.442 | 0.142 | 0.183 |
| E5 | 0.578 | **0.725** | 0.475 | 0.218 | 0.211 |
| E6 | 0.546 | **0.814** | 0.403 | 0.168 | 0.115 |
| E7 | 0.642 | **0.823** | 0.500 | 0.147 | 0.172 |
| E8 | 0.582 | **0.733** | 0.489 | 0.134 | 0.141 |
| E9 | 0.481 | **0.725** | 0.436 | 0.165 | 0.203 |
| L1 | 0.560 | 0.427 | **0.727** | 0.179 | 0.212 |
| L2 | 0.440 | 0.347 | **0.716** | 0.136 | 0.180 |
| L3 | 0.500 | 0.448 | **0.783** | 0.211 | 0.182 |
| L4 | 0.563 | 0.452 | **0.814** | 0.204 | 0.153 |
| L5 | 0.556 | 0.427 | **0.797** | 0.214 | 0.209 |
| L6 | 0.587 | 0.447 | **0.770** | 0.194 | 0.161 |
| L7 | 0.558 | 0.382 | **0.753** | 0.104 | 0.131 |
| L8 | 0.562 | 0.501 | **0.769** | 0.190 | 0.154 |
| L9 | 0.511 | 0.389 | **0.751** | 0.182 | 0.149 |
| L10 | 0.570 | 0.438 | **0.781** | 0.100 | 0.090 |
| L11 | 0.555 | 0.502 | **0.803** | 0.200 | 0.158 |
| L12 | 0.558 | 0.494 | **0.785** | 0.209 | 0.149 |
| SHS1 | 0.213 | 0.212 | 0.225 | **0.920** | 0.569 |
| SHS2 | 0.102 | 0.105 | 0.118 | **0.843** | 0.458 |
| SHS3 | 0.207 | 0.207 | 0.235 | **0.899** | 0.468 |
| SWLS1 | 0.202 | 0.237 | 0.205 | 0.468 | **0.860** |
| SWLS2 | 0.143 | 0.181 | 0.152 | 0.417 | **0.831** |
| SWLS3 | 0.170 | 0.183 | 0.177 | 0.570 | **0.884** |
| SWLS4 | 0.153 | 0.194 | 0.156 | 0.424 | **0.763** |
